# Experimental Myocardial Infarction Elicits Time-Dependent Patterns of Vascular Hypoxia in Peripheral Organs and in the Brain

**DOI:** 10.3389/fcvm.2020.615507

**Published:** 2021-01-27

**Authors:** Hélène David, Aurore Ughetto, Philippe Gaudard, Maëlle Plawecki, Nitchawat Paiyabhroma, Emma Zub, Pascal Colson, Sylvain Richard, Nicola Marchi, Pierre Sicard

**Affiliations:** ^1^INSERM, CNRS, Université de Montpellier, PHYMEDEXP, Montpellier, France; ^2^Department of Anesthesiology and Critical Care Medicine, Arnaud de Villeneuve Hospital, CHU Montpellier, Montpellier, France; ^3^CHU Lapeyronie, Département de Biochimie, Montpellier, France; ^4^Cerebrovascular and Glia Research, Department of Neuroscience, Institute of Functional Genomics (UMR 5203 CNRS – U 1191 INSERM, University of Montpellier), Montpellier, France; ^5^Montpellier University, INSERM, CNRS, Institut de Génomique Fonctionnelle, Montpellier, France; ^6^IPAM, BioCampus Montpellier, CNRS, INSERM, Université de Montpellier, Montpellier, France

**Keywords:** myocardial infarction, cardiogenic shock, hypoxia, cerebrovasculature, photoacoustic imaging, microcirculation, pericytes

## Abstract

**Aims:** Microvascular alterations occurring after myocardial infarction (MI) may represent a risk factor for multi-organ failure. Here we used *in vivo* photoacoustic (PA) imaging to track and define the changes in vascular oxygen saturation (sO_2_) occurring over time after experimental MI in multiple peripheral organs and in the brain.

**Methods and Results:** Experimental MI was obtained in BALB/c mice by permanent ligation of the left anterior descending artery. PA imaging (Vevo LAZR-X) allowed tracking mouse-specific sO_2_ kinetics in the cardiac left ventricular (LV) anterior wall, brain, kidney, and liver at 4 h, 1 day, and 7 days post-MI. Here we reported a correlation between LV sO_2_ and longitudinal anterior myocardial strain after MI (*r* = −0.44, *p* < 0.0001, *n* = 96). Acute LV dysfunction was associated with global hypoxia, specifically a decrease in sO_2_ level in the brain (−5.9%), kidney (−6.4%), and liver (−7.3%) at 4 and 24 h post-MI. Concomitantly, a preliminary examination of capillary NG2DsRed pericytes indicated cell rarefication in the heart and kidney. While the cardiac tissue was persistently impacted, sO_2_ levels returned to pre-MI levels in the brain and in peripheral organs 7 days after MI.

**Conclusions:** Collectively, our data indicate that experimental MI elicits precise trajectories of vascular hypoxia in peripheral organs and in the brain. PA imaging enabled the synchronous tracking of oxygenation in multiple organs and occurring post-MI, potentially enabling a translational diagnostic modality for the identification of vascular modifications in this disease setting.

## Introduction

Tissular hypoxia following end-organ hypoperfusion after myocardial infarction (MI) is associated with cardiogenic shock incidence and elevated mortality (1). Despite the use of early coronary revascularization and the advances in cardiogenic shock treatment, the inability to rapidly restore regional tissular normoxia represents a critical factor impacting MI prognosis (2). Monitoring, as early as possible, organ-specific dynamics of hypoxia represents a candidate strategy to guide treatments and to timely identify high-risk patients (3). Currently, serum biomarkers like lactate are used to gauge global tissue perfusion. However, lactate is not specific to an organ, whereas microvascular perfusion is naturally heterogeneous (4). Recently, novel technologies are emerging to assess microcirculation at the patient's bedside (5). Continuous real-time monitoring of specific organ oxygenation is clinically relevant, and using near infrared spectroscopy (NIRS) was proposed to monitor hypoxia and to enable a guided therapy in shock patients (6). However, the spatio-temporal resolution of NIRS is proven to be insufficient (7). As a result, our understanding of the patterns of multi-organ hypoxia elicited in response to MI remains incomplete.

Overcoming current technological and knowledge stumbling blocks, we here used photoacoustic imaging (PAI), a hybrid technology based on the optical excitation of endogenous molecules by laser pulses, resulting in thermoelastic waves detected by high-resolution ultrasound (US) receivers (8). We tested the hypothesis that high-resolution PAI could detect time-dependent and synchronous organ-specific patterns of hypoxia elicited by experimental MI. We successfully generated a fusion of anatomical and molecular imaging data specifically exploiting hemoglobin to create a real-time vascular oxygen saturation (sO_2_) mapping of multiple organs. We report the varying hypoxia trajectories simultaneously unfolding in the heart, brain, kidney, and liver hours to days after MI. Finally, we performed an exploratory, qualitative examination of vascular cell modifications associated to post-MI hypoxia, focusing on NG2DsRed pericytes.

## Materials and Methods

### Experimental Model

**Figure 1:**
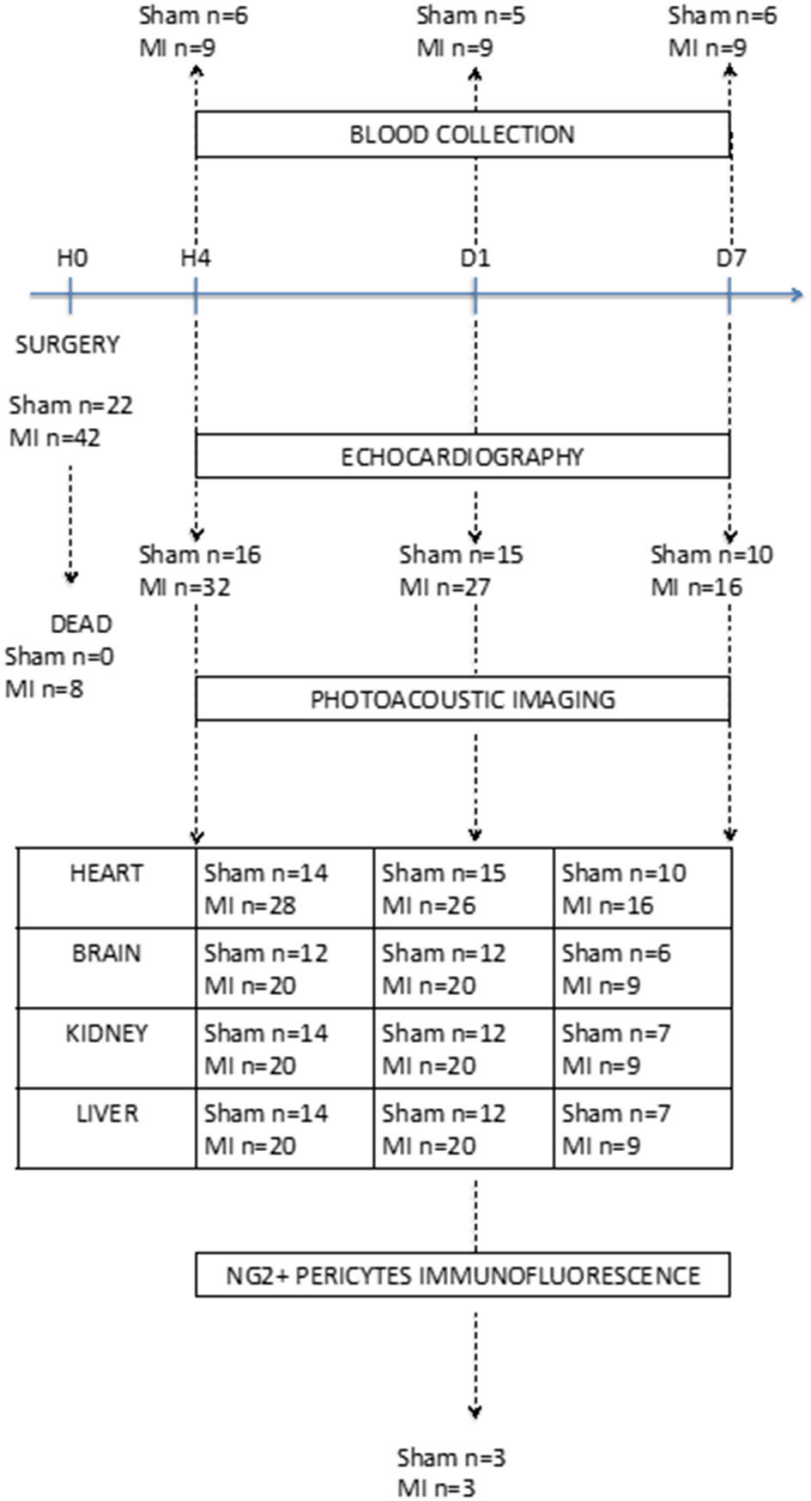
Flow chart showing the timeline and number of mice used in each group in the applied study design. Echocardiography and photoacoustic imaging were performed at 4 h, 1 day, and 7 days after surgery (*n* = 7–28 per group). Mice were euthanized at three time points (4 h, day 1, and day 7) for blood collection (*n* = 5–9 per group). For the NG2DsRed pericyte immunofluorescence experiment, mice were sacrificed 24 h after surgery (*n* = 3 per group).

Seventy male BALB/c mice of 8 weeks, weighing 25–30 g, were randomly assigned to either sham surgery (sham *n* = 25) or MI group (*n* = 45) defined by left anterior descending coronary artery (LAD) ligation performed as described (9). Eight mice died in the MI group (18%) during the 7-day follow-up period (Figure 1). No mice died in the sham group. All experimental procedures were conducted in accordance with the European Union Laboratory Animal Care Rules (2010/63/EU Directive) and were approved by the Animal Care and Use Committee of the University of Montpellier (no. 13129-2018011910135259). Based on previous statistical analysis, we used a minimum of animals in order to comply with the 3Rs principles. The mice were housed in a pathogen-free facility and handled in accordance with the principles and procedures outlined in the ARRIVE guidelines (10).

### Echocardiography and Speckle Tracking Analysis

High-resolution echocardiography (Vevo-3100, VisualSonics/Fujifilm, with a 40-MHz MX550D ultrasound probe) was performed to assess left ventricular (LV) function (ejection fraction and longitudinal strain) at 4 h, 1 day, and 7 days after surgery according to the guidelines of the American Physiological Society as applied to mice after MI and by the European Society of Cardiology Working Group on Myocardial Function (11, 12). Echocardiography was performed under general anesthesia by 2% isoflurane inhalation, and the body temperature was 37°C. The ECG and respiratory rate were monitored. B-mode parasternal short-axis multiple views from apex to base were recorded to measure the LV ejection fraction (LVEF). After tracing endocardial end-diastolic and end-systolic areas, LV volumes were calculated by Simpson's method of disks, and LVEF was determined from the following formula: (end-diastolic LV volume - end-systolic LV volume)/(end-diastolic LV volume). Global and regional longitudinal strain analysis was performed under the parasternal long-axis view. Offline image analysis was performed using dedicated VisualSonics Vevolab 3.1.0 and Vevostrain software.

### Photoacoustic Imaging

The Vevo LAZR-X (VisualSonics/Fujifilm) system was used in this study to generate the photoacoustic (PA) images in association with Vevo3100. PA images were obtained at 750- and 850-nm excitation wavelengths, with the array transducer of 20 MHz central frequency (MX250), due to maximal absorption of oxy- and deoxyhemoglobin at these wavelengths using the oxy-hemo mode to determine sO_2_. For the PA signal acquisition, the gain was set to 34 dB with 2D gain of 22 dB. A B-mode was also performed for colocalization of photoacoustic signals. Measurements were realized at 4 h, 1 day, and 7 days after surgery. In order to increase temporal and spatial resolution for myocardial anterior wall sO_2_ measurement, we combined PA imaging with ECG-gated kilohertz visualization. Calculation of sO_2_ was achieved offline with the VevoLAB software by selecting areas of interest (myocardial anterior wall, brain, liver, and kidney) in B-mode.

### Blood Sample and Plasma Biomarker Dosage

After sacrificing the mice by dislocation, direct intracardiac puncture by thoracotomy was performed using 21 G (0.8 × 16 mm) needle (BD Microlance®) to obtain a maximum volume of blood as possible. Blood was collected in 2-ml heparinized tubes and centrifuged at 8,000 rpm at 5°C for 8 min. The supernatants were decanted, frozen in liquid nitrogen, and preserved at −80°C until the time of assay. The plasma concentrations of lactate, creatinine, alanine amino transferase (ALAT), and aspartate amino transferase (ASAT) were determined using a Cobas 8000 analyzer (Roche Diagnostics, Meylan, France). The creatinine levels were measured with an IDMS traceable enzymatic method. ALAT and ASAT concentrations were performed according to the International Federation of Clinical Chemistry, without pyridoxal phosphate activation.

### NG2DsRed Pericyte Immunofluorescence and Quantification

We used an additional set of C57BL/6j (13) to visualize and quantify NG2DsRed pericytes in the heart, brain, kidney, and liver. Tissue sections (30 μm) were obtained using a vibratome and rinsed with phosphate-buffered saline (PBS). Blood–brain barrier damage was assessed as described previously (14). Briefly, a solution containing sodium fluorescein (F6377, Sigma) was prepared immediately before the experiments (50 mg/ml in PBS). Each mouse was injected (i.v., 50 μl for a 25-g mouse) and sacrificed 2 h later. The mice were perfused intracardially using cold PBS, and the brains were post-fixed in 4% paraformaldehyde for 2 days.

Fluorescent images (DAPI, fluorescein, and NG2DsRed) were obtained as mosaic maps (×10) of whole tissue sections of the heart, brain, kidney, and liver. The images were acquired using a Zeiss epifluorescence microscope. The images were modulated in red, green, and blue RGB color/RGB Zstack format type. Fluorescence signal area was calculated for each image, expressed as a percentage of total area, and quantified using Fiji/ImageJ 1.52p software.

### Statistical Analysis

Results are expressed as mean ± SEM. Single comparisons between two independent means were made using Student's *t* test or Mann–Whitney test as appropriate. The correlations between echocardiography parameters, plasma biomarkers, and PAI were statistically evaluated using Pearson's correlation tests. Statistical significance was considered at *p* < 0.05; the *p* values were two-tailed. Analyses were performed using GraphPad Prism 6 software.

## Results

### Left Ventricular Dysfunction Correlated With Myocardial sO_2_ Level

**Figure 2:**
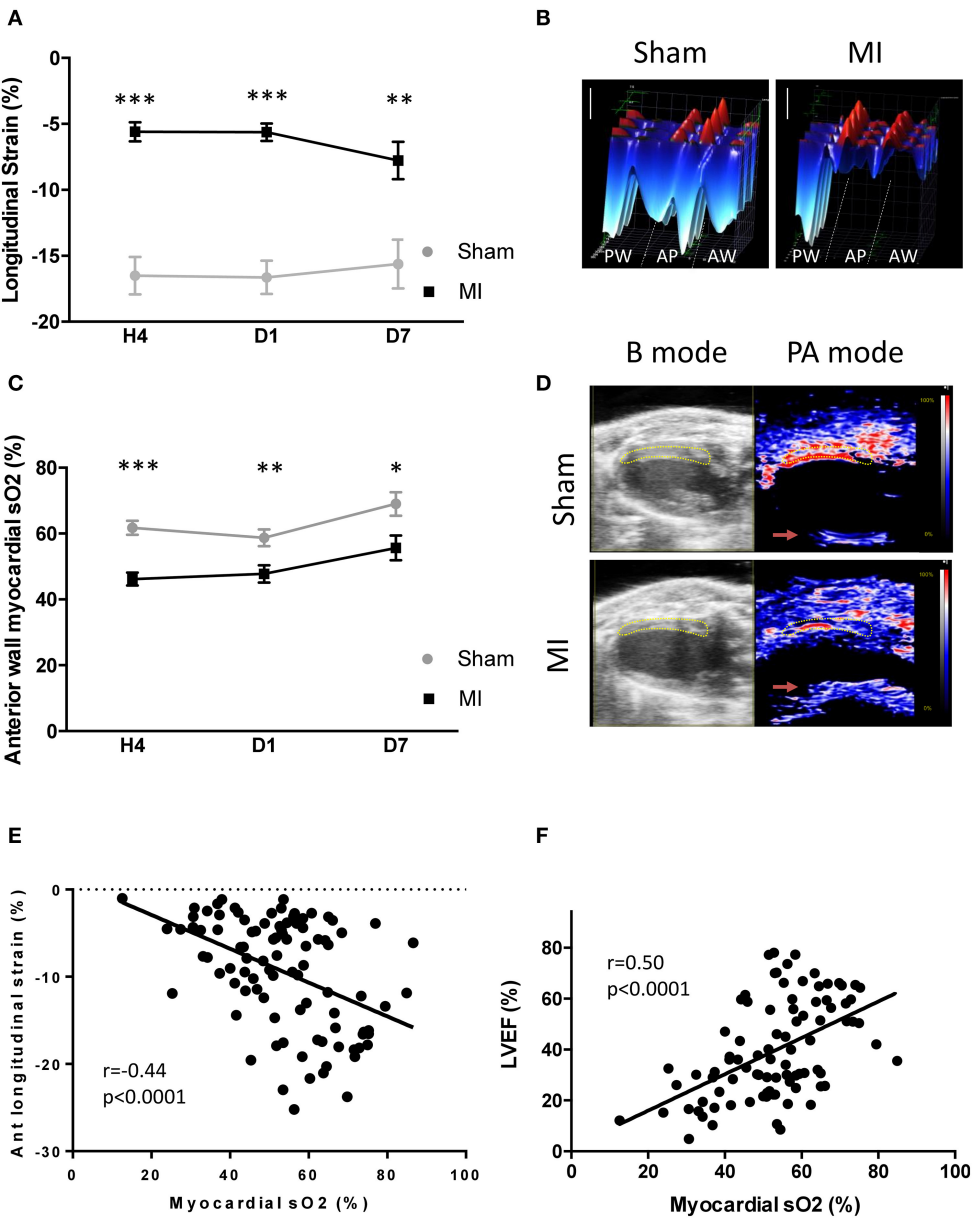
Cardiac function and myocardial oxygen saturation (sO_2_) after acute myocardial infarction. **(A)** Global longitudinal strain analysis at 4 h, day 1, and day 7 after surgery in the sham and myocardial infarction (MI) groups. **(B)** Representative 3D myocardial strain in sham and MI animals. Scale vertical bar: 10%. **(C)** Variation of myocardial anterior wall sO_2_ in the sham and MI groups at 4 h (*n* = 14 and 28 mice, respectively), day 1 (*n* = 15 and 26 mice, respectively), and day 7 (*n* = 10 and 16 mice, respectively) after surgery. **(D)** Representative B mode echocardiography parasternal long-axis view to define the left ventricular anterior wall (yellow area) and photoacoustic mode with color scaling to show areas of high oxygen saturation in red and low saturation in blue in sham and MI mice. The red arrows shows unspecific PA reverberation artifacts. **(E)** Pearson correlation between anterior wall myocardial sO_2_ and left ventricular anterior wall longitudinal strain (*n* = 96). **(F)** Pearson correlation between anterior wall myocardial sO_2_ and left ventricular ejection fraction (*n* = 107). Results are expressed as mean ± SEM. Experimental groups were compared using Student's *t* test or Mann–Whitney test as appropriate. *p < 0.05; **p < 0.01; ***p < 0.001 sham vs. MI.

LV function, assessed by global longitudinal strain analysis (Figures 2A,B), was reduced 4 h post-MI (*p* < 0.001). These alterations persisted at 1 day (*p* < 0.001) and 7 days (*p* = 0.01) post-MI. The LVEF followed similar kinetics (Supplementary Figure 1A), while the heart rate (Supplementary Figure 1B) was comparable between groups over time.

Next, we tested the ability of PAI to detect sO_2_ changes in the anterior wall after LAD occlusion (Figures 2C,D). A decrease in sO_2_ level occurred in the LV anterior wall t 4 h (sham 61.8 ± 2.1%, *n* = 14; MI: 44.3 ± 2.2% *n* = 28; *p* < 0.001), 1 day (sham: 57.3 ± 2.6%, *n* = 15; MI: 48.9 ± 2.8%, *n* = 26; *p* < 0.01), and 7 days (sham: 69.0 ± 3.5%, *n* = 10; MI: 57.6 ± 4.0%, *n* = 16; *p* < 0.05) after LAD ligation (Figure 2C). A correlation between LV sO_2_ and LV anterior wall longitudinal strain was found at 4 h (*r* = −0.43, *p* < 0.01; *n* = 38) (Supplementary Figure 2A), at day 1 (*r* = −0.41, *p* < 0.01; *n* = 36) (Supplementary Figure 2B), and at day 7 (*r* = −0.48, *p* = 0.02; *n* = 22) (Supplementary Figure 2C). This was also verified when we pooled all data together (Figure 2E). In addition, a correlation between LV sO_2_ and EF existed in the pooled data (Figure 2F) at 4 h (*r* = −0.50, *p* < 0.001; *n* = 42) (Supplementary Figure 3A) and at day 1 (*r* = −0.51, *p* < 0.001, *n* = 41) (Supplementary Figure 3B), but not at day 7 (*r* = 0.27, *p* = 0.19; *n* = 24) (Supplementary Figure 3C). Taken together, these results indicate that PAI is able to detect and track hypoxia in the LV anterior wall after MI.

### Vascular Hypoxia in Peripheral Organs and the Brain After Acute MI

**Figure 3:**
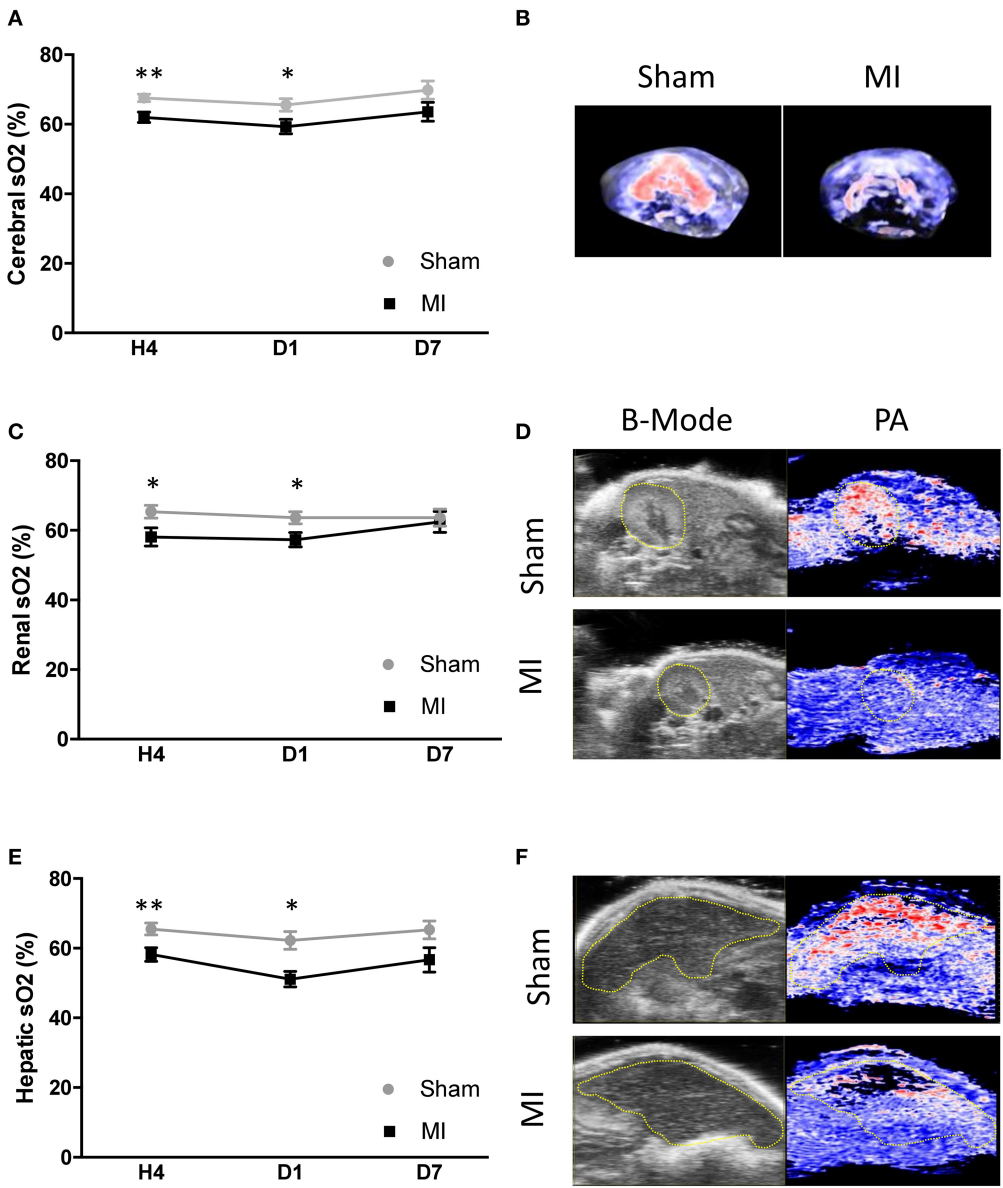
Cerebral and peripheral organ oxygen saturation (sO_2_) level assessed by photoacoustic secondary to myocardial infarction (MI). **(A)** Variation of cerebral sO_2_ in the sham and MI groups at 4 h (*n* = 12 and 20 mice, respectively), day 1 (*n* = 12 and 20 mice, respectively), and day 7 (*n* = 6 and 9 mice, respectively) after surgery. **(B)** Representative 3D cerebral photoacoustic images with color scaling to show areas of high oxygen saturation in red and low saturation in blue in sham and MI mice. **(C)** Variation of renal sO_2_ in the sham and MI groups at 4 h (*n* = 14 and 20 mice, respectively), day 1 (*n* = 12 and 20 mice, respectively), and day 7 (*n* = 7 and 9 mice, respectively) after surgery. **(D)** Representative B mode abdominal ultrasound to define the kidney (yellow area) and photoacoustic mode with color scaling to show areas of high oxygen saturation in red and low saturation in blue in sham and MI mice. **(E)** Variation of hepatic sO_2_ in the sham and MI groups at 4 h (*n* = 14 and 20 mice, respectively), day 1 (*n* = 12 and 20 mice, respectively), and day 7 (*n* = 7 and 9 mice, respectively) after surgery. **(F)** Representative B mode abdominal ultrasound to define the liver (yellow area) and photoacoustic mode with color scaling to show areas of high oxygen saturation in red and low saturation in blue in sham and MI mice. Results are expressed as mean ± SEM. Experimental groups were compared using Student's *t* test or Mann–Whitney test as appropriate. *p < 0.05; **p < 0.01; sham vs. MI.

**Figure 4:**
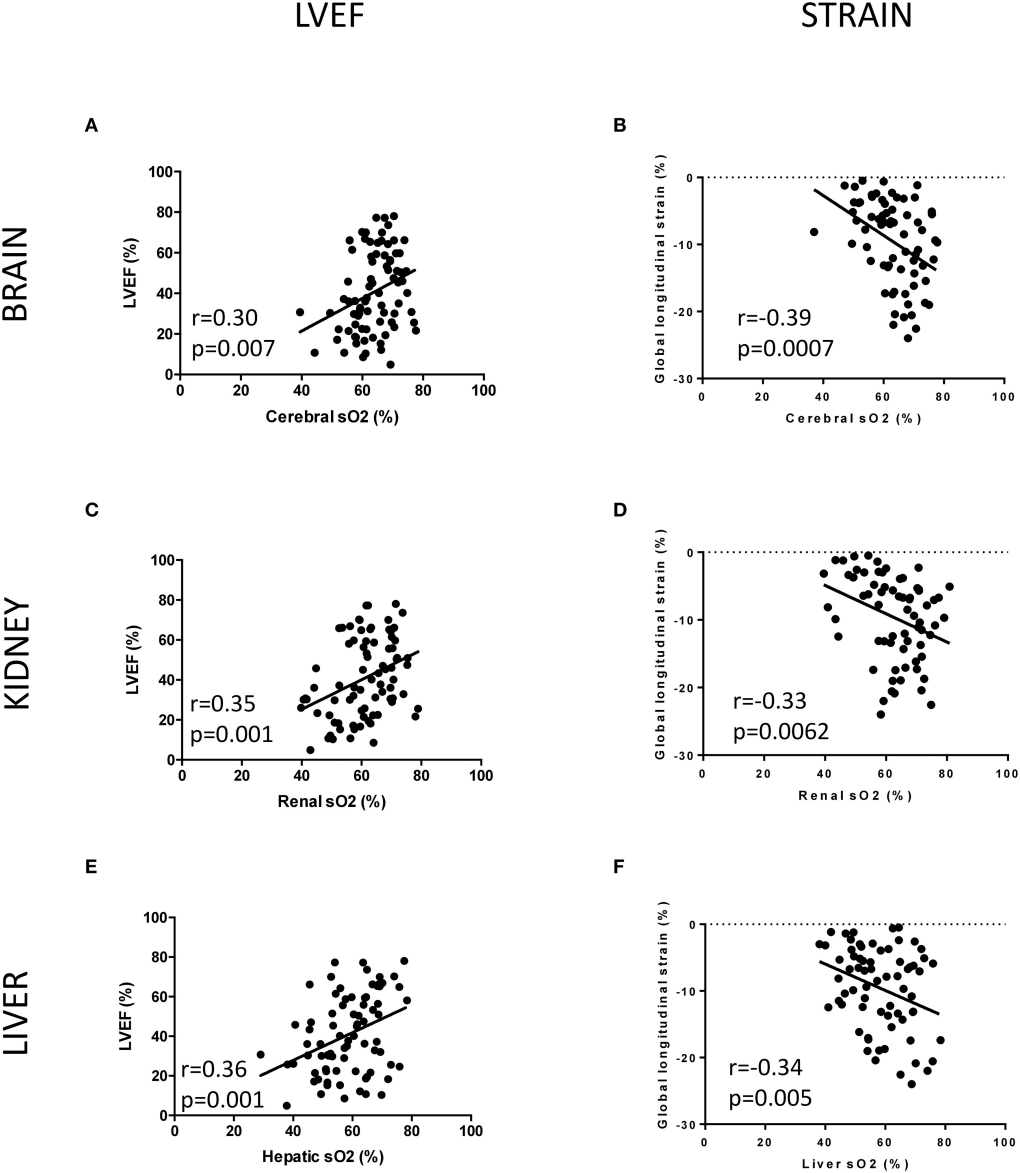
Pearson correlation between left ventricular ejection fraction (LVEF) or global longitudinal strain and sO_2_ organ mapping. **(A)** Pearson correlation between LVEF and cerebral sO_2_ (*n* = 80). **(B)** Pearson correlation between global longitudinal strain and cerebral sO_2_ (*n* = 69). **(C)** Pearson correlation between LVEF and renal sO_2_ (*n* = 81). **(D)** Pearson correlation between global longitudinal strain and renal sO_2_ (*n* = 69). **(E)** Pearson correlation between LVEF and hepatic sO_2_ (*n* = 81). **(F)** Pearson correlation between global longitudinal strain and hepatic sO_2_ (*n* = 69).

Subsequentially, we tested the hypothesis that acute cardiac dysfunction after MI would induce multi-organ vascular hypoxia. We combined high-resolution ultrasound and PAI to localize and quantify sO_2_ in the brain, kidney, and liver in sham and MI animals. Examples of high-resolution US/PAI images are provided in Figures 3B,D,F. Photoacoustic mapping outlined an early decrease in sO_2_ level after MI, compared to sham, by −5.9% in the whole brain (Figures 3A,B), −6.4% in the kidney (Figures 3C,D), and −7.3% in the liver (Figures 3E,F) at 4 h post-MI. Next, we tested the hypothesis that the level of cardiac dysfunction after MI, measured either by LVEF or global longitudinal strain, would correlate with vascular oxygen desaturation of specific organs. A significant correlation existed between brain sO_2_ level and LVEF (Figure 4A) and global longitudinal strain (Figure 4B). LVEF and global longitudinal strain were significantly correlated with renal sO_2_ level (Figures 4C,D). In addition, we observed a positive correlation between cardiac dysfunction and liver sO_2_ level (Figures 4E,F). Collectively, these results outline the global and varying oxygen dynamics occurring in response to MI, correlating to cardiac dysfunction.

### Hypoxic Tissue Mapping Positively Correlated With Blood Biomarkers of Organ Dysfunction

**Figure 5:**
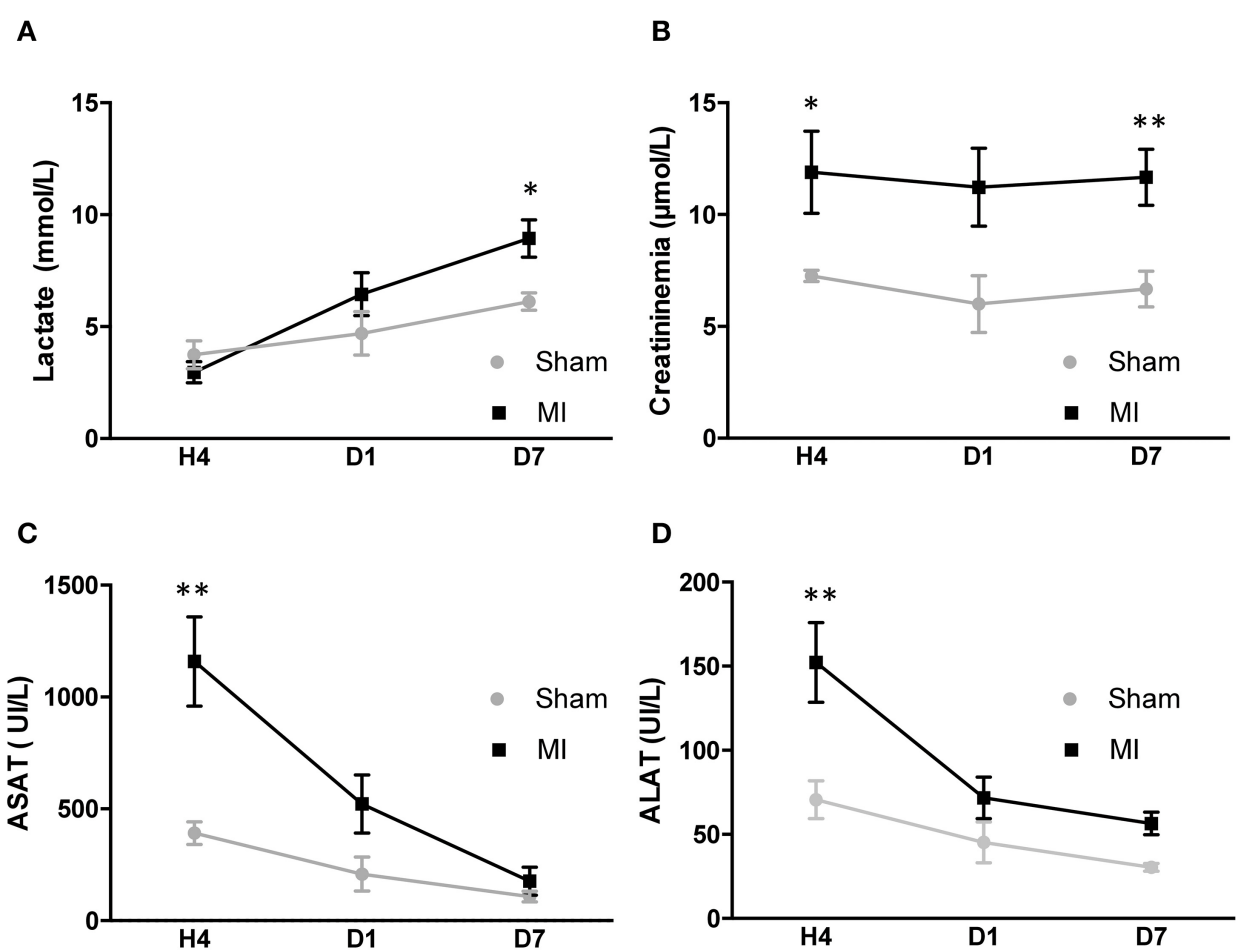
Plasmatic biomarker kinetics after acute myocardial infarction (MI) in mice. **(A)** Variation of lactate in the sham and MI groups (*n* = 6 and 9 mice, respectively) at 4 h, day 1, and day 7 after surgery. **(B)** Variation of creatinemia in the sham and MI groups (*n* = 6 and 9 mice, respectively) at 4 h, day 1, and day 7 after surgery. **(C)** Variation of ASAT in the sham and MI groups (*n* = 6 and 9 mice, respectively) at 4 h, day 1, and day 7 after surgery. **(D)** Variation of alanine amino transferase in the sham and MI groups (*n* = 6 and 9 mice, respectively) at 4 h, day 1, and day 7 after surgery. Results are expressed as mean ± SEM. Experimental groups were compared using Mann–Whitney test. *p < 0.05; **p < 0.01; sham vs. MI.

The circulatory levels of clinically relevant organ dysfunction biomarkers (lactate, creatinine, ASAT, and ALAT) were measured at each time point (Figure 5). The kinetic of lactate showed a significant increase at 7 days post-MI (Figure 5A). Renal function was assessed by measuring the creatinine level. Creatinemia was increased at 4 h post-MI compared to sham animal and did not return to normal level at 7 days post-MI (Figure 5B).

**Figure 6:**
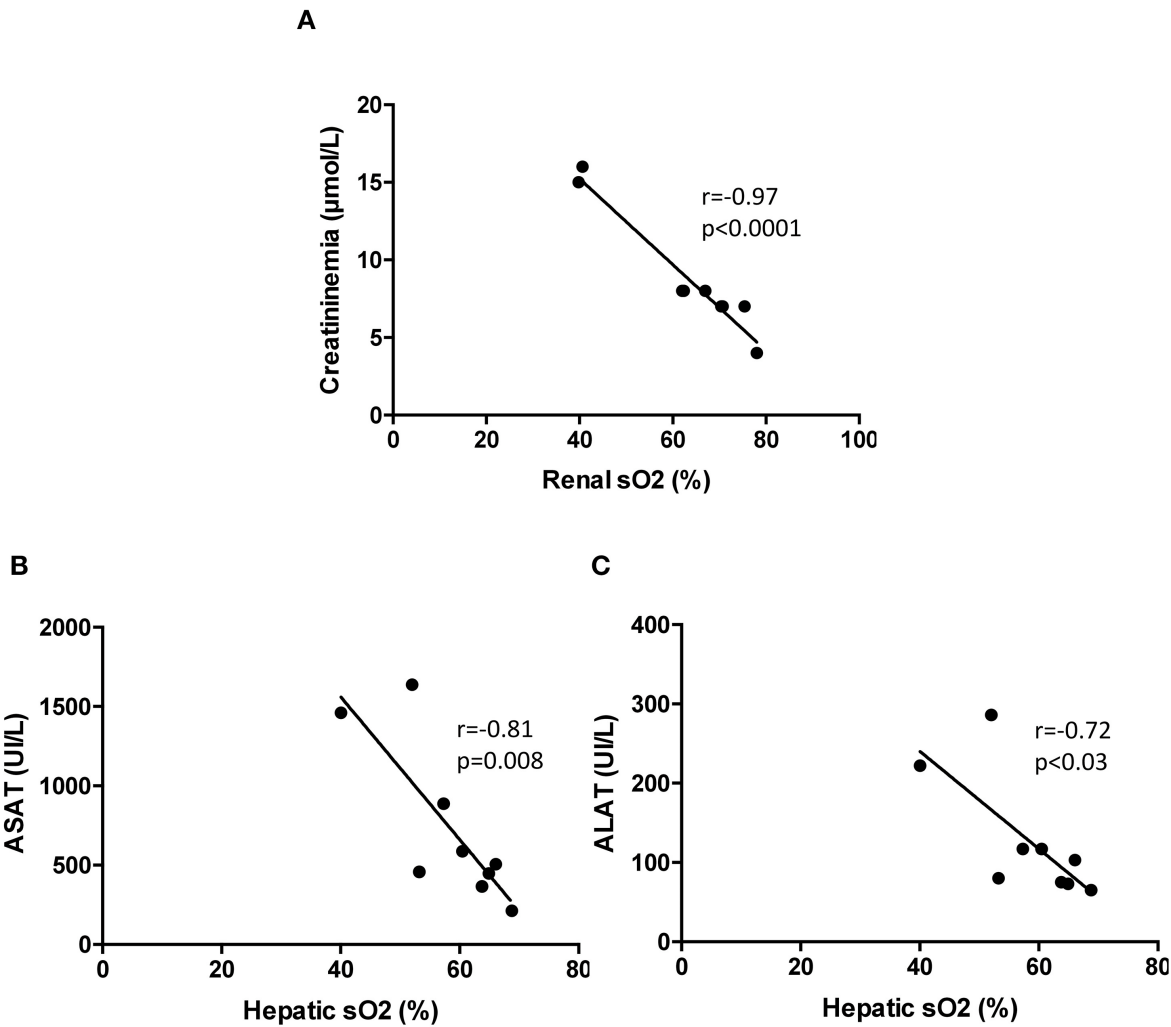
Correlations between plasmatic biomarkers and organ sO_2_ mapping at 4 h after surgery in mice. **(A)** Pearson correlation between renal sO_2_ and creatininemia (*n* = 9). **(B)** Pearson correlation between hepatic sO_2_ and aspartate amino transferase (*n* = 9). **(C)** Pearson correlation between hepatic sO_2_ and alanine amino transferase (*n* = 9). sO_2_, oxygen saturation assessed by photoacoustic imaging.

The blood levels of hepatic cytotoxic biomarkers ASAT and ALAT were elevated 4 h post-MI as compared to sham and then decreased at day 1, returning to baseline levels at 7 days (Figures 5C,D). The kinetic of creatinine (Figure 6A), ASAT (Figure 6B) and ALAT (Figure 6C) correlated to kidney and liver modifications of sO_2_ at 4 h. However, no correlation existed between the organs' sO_2_ and lactate (Supplementary Figure 6). In general, LV dysfunction post-MI is associated with an increase in biomarkers of organ dysfunction.

### Histological Signs of Vascular Dysfunction: A Preliminary Focus on Capillary Pericytes

**Figure 7:**
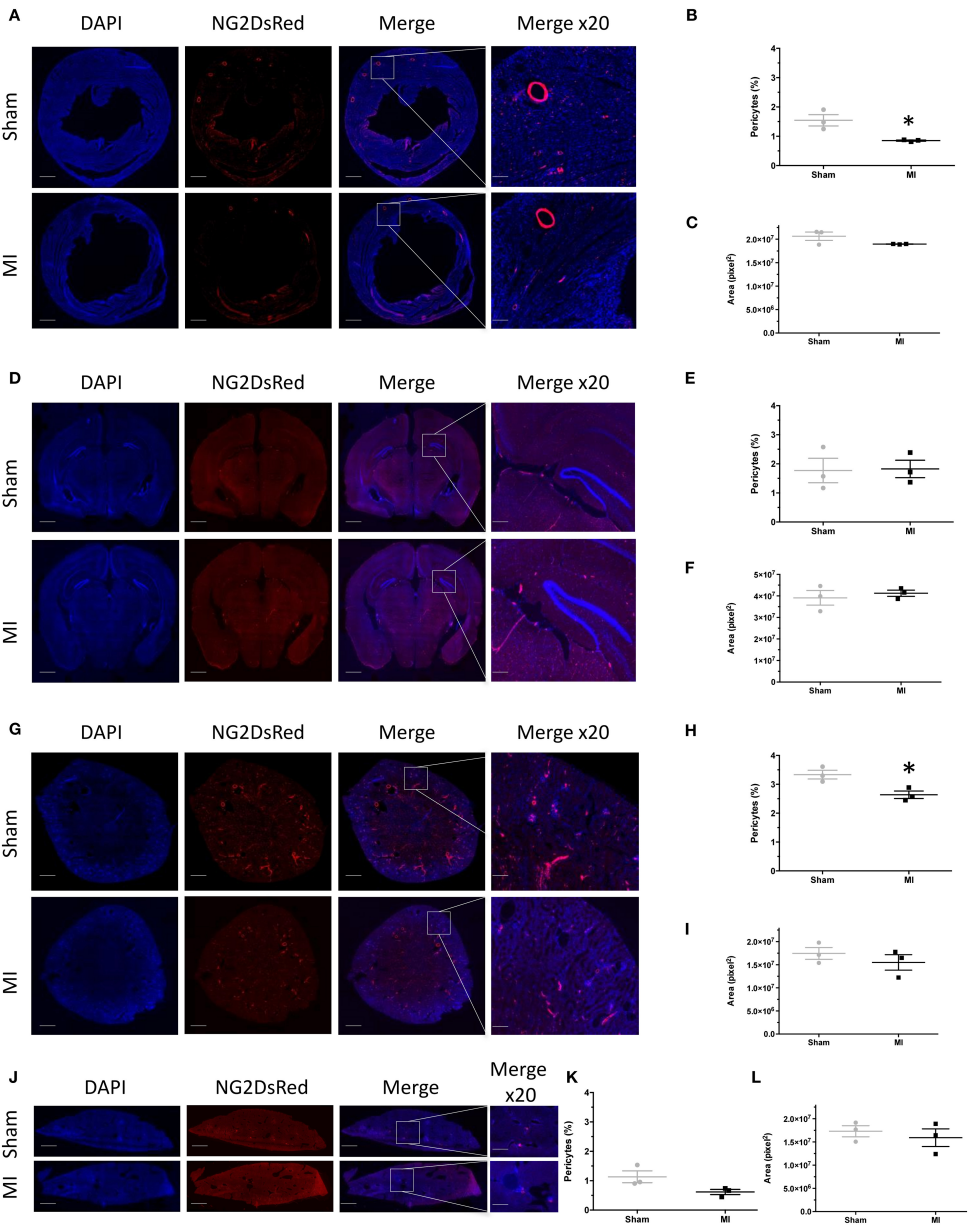
NG2DsRed pericyte fluorescence and comparison between sham and myocardial infarction (MI). **(A)** Representative myocardial fluorescence images of nuclei (DAPI), NG2DdRed pericytes in sham and MI 24 h after surgery. **(B)** Myocardial fluorescence intensity quantification of NG2DdRed pericytes in sham and MI. **(C)** Myocardial area measurement in each group. **(D)** Representative brain fluorescence images of nuclei (DAPI), NG2DdRed pericytes in sham, and MI 24 h after surgery. **(E)** Brain fluorescence intensity quantification of NG2DdRed pericytes in sham and MI. **(F)** Brain area measurement in each group. **(G)** Representative renal fluorescence images of nuclei (DAPI), NG2DdRed pericytes in sham, and MI 24 h after surgery. **(H)** Renal fluorescence intensity quantification of NG2DdRed pericytes in sham and MI. **(I)** Renal area measurement in each group. **(J)** Representative hepatic fluorescence images of nuclei (DAPI), NG2DdRed pericytes in sham, and MI 24 h after surgery. **(K)** Hepatic fluorescence intensity of NG2DdRed pericytes in sham and MI. **(L)** Hepatic area measurement in each group. Sham: *n* = 3 and myocardial infarction MI: *n* = 3. Results are expressed as mean ± SEM. Experimental groups were compared using Student's *t* test. *p < 0.05 sham vs. MI. Scale: 100 μm.

Pericytes are a type of mural cell partaking to structural and functional vascular properties. Pericytes are extremely sensitive to hypoxia as it elicits during peripheral and brain pathologies (15, 16). Here we specifically used C57BL/6j NG2DsRed mice to histologically assess pericyte modifications elicited after acute MI in cardiac, cerebral, renal, and hepatic tissues (Figure 7). Our initial explorations unveiled a decrease in cardiac and renal NG2DsRed pericytes 24 h post-MI as compared to sham (Figures 7A–C,G–I). Under these experimental conditions, we did not observe global cell modifications in the brain and the liver (Figures 7D–F,J–L). Our results indicate that LAD ligation induces global, but transient, hypoxia that is sufficient to promote pericyte modifications in the heart and kidney.

## Discussion

Our pre-clinical study discloses the multi-organ and brain trajectories of vascular oxygenation unfolding *in vivo* consequent to experimental MI. We report here that: (i) LAD ligation in mice induced an early and transient decrease of sO_2_ in the brain, kidney, and liver, (ii) oxygenation was significantly correlated with LV dysfunction as assessed by regional strain analysis and LVEF after acute MI, (iii) sO_2_ level was correlated with blood biomarkers (creatinine, ASAT, and ALAT), and (iv) pericytes could represent one possible entry point for further studying the multi-organ vascular dysfunctions elicited in this disease settings. In summary, the use of non-invasive PAI enabled a robust and time-dependent sO_2_ mapping in peripheral organs and the brain after MI.

### Multiple Organ Dysfunction Post-MI: Experimental and Clinical Values

The pathophysiology behind multiple-organ injury during acute MI is poorly understood as innovative imaging modalities for vascular read-outs lagged behind. Primarily, we successfully validated PAI by detecting oxygenation decrease in the anterior wall following myocardial ischemia. Importantly, the anterior wall remodeling process after MI is dynamic. After cardiomyocyte necrosis, fibroblasts and immune cells are infiltrated into the infarction and border area within the first 7 days after MI. Extensive endothelial cell plasticity was recently described as a novel circulatory system in which new vessels develop from the endocardium of the left ventricle to perfuse the hypoxic area and recover damaged cardiomyocytes after MI (17). We assume that the increased oxygen saturation of the anterior wall is due to this mechanism.

To our knowledge, this is the first preclinical study showing a correlation between non-cardiac oxygenation level and LV dysfunction after acute MI. Our results are coherent with the notion of pericyte loss in response to hypoxia, mechanical stress (18), or increased oxidative stress (19), all occurring during and after MI (20). Hypoxic signaling pathways lead to contraction of the pericytes, causing constriction of the capillaries (21–23), followed by death of pericytes in rigor which prevents subsequent capillary dilation (24). These mechanisms can induce vessel permeability, a remodeling process, and a rapid decay in capillary density (25).

Here we report brain desaturation occurring as early as at 4 h post-MI and persisting at 1 day, before returning to control levels at 7 days. The transient oxygen desaturation occurring in these experimental conditions did not impact the gross morphology of mural cells. As a corollary, cerebrovascular permeability was preserved at day 1 after MI as indicated by the lack of intravenously injected fluorescein into the brain parenchyma (data not shown). However, these explorations were very limited, and they should be integrated with more in-depth analyses. Interestingly, recent data indicated that mild and transitory cerebrovascular hypoxia, detected by PAI, may contribute to long-term and negative neurological sequelae (14). A recent translational study demonstrated, using non-invasive positron emission tomography, that MI leads to a late neuro-inflammatory response (26). Thus, cerebral hypoxia after MI could trigger neuroinflammation and possibly neurological dysfunction in the long term.

### PA Technology as a New Modality to Define Hypoxia Trajectories

Alterations in the microcirculation are a pathophysiological hallmark in cardiac failure, especially in cardiogenic shock, and a central feature of organ failure. The coexistence of cardiac dysfunction and multi-organ failure are recognized as a marker of poor prognosis in cardiogenic shock patients (1). Assessment of the peripheral microcirculation and hypoxic status in acute heart failure patients is recommended in the current guidelines (27). Here we integrated and cross-compared our PAI results with biomarkers of renal dysfunction and liver cytolysis. A correlation between biomarker levels and sO_2_ was established for creatinine, ASAT, and ALAT. However, lactate elevation in this mouse model of MI failed to correlate with the measurement of PAI oxygenation. This discrepancy could be explained by the fact that, in our mouse MI model, the oxygenation levels of the major organ levels were only 7% lower than sham. Notwithstanding, the currently used blood biomarkers are not organ specific, and hypoperfusion is not the only underlying cause for the elevation in lactate, but reveals a much more holistic picture for patients (28).

In our study, the fusion of anatomical and molecular imaging reveals mild and early hypoxic status in individual organs with great precision. An array of techniques can be used to evaluate microcirculatory modifications in disease settings. Direct methods include capillaroscopy (nail-fold), laser Doppler, and intravital microscopy (7). Indirect indicators are blood pressure, central venous pressure, capillary refill, mottling score, mixed-venous and central venous oxygen saturation (SvO_2_/ScvO_2_), contrast ultrasound and echocardiography, measurement for O_2_ saturation with NIRS, gas tomography, veno-arterial CO_2_ gradient, and vascular occlusion test with NIRS or Doppler laser (7). Except for SvO_2_/ScvO_2_ and lactate, none of these techniques are routinely used in clinical practice. They are not sufficient to guide therapy in critically ill patients because of the lack of organ specificity, the limited availability in clinical practice, offline analysis, and the lack of automated analysis system without any specific scoring systems (29). The combination of high-resolution US and PA systems allowed a clear identification of anatomical structures and molecular imaging and provided valuable functional information about the degree of tissue oxygenation. Gerling et al. (30) assessed hypoxia and tumor blood flow in murine models of pancreatic and colon cancer and reported a positive correlation of PAI data with immuno-histochemical signs of hypoxia.

PA imaging technology has demonstrated great potential for clinical translation. Recently, PA was used on pretransplant human kidney to assess collagen and fibrosis content in order to determine organ quality before transplantation (31). In addition, early diagnosis of breast and prostate cancer (32–34) and melanoma (35) and detection of tumor metastases (36) were obtained using PA imaging directly in patients. Despite the major advantages to use this non-ionizing and non-invasive technique to detect molecular and anatomical defects in patients, some technical limitation remains a brake for its routine clinical use. PA imaging performance is limited by several technical and anatomical issues. One limitation of current PAI systems is the limited penetration depth. In our study, we combined nanosecond laser with a high-frequency ultrasound probe (20 MHz), resulting in 2-cm-depth PAI images. The use of a lower-frequency US probe and higher laser energy is a realistic option to improve applicability. We believe that with continued technological development and higher laser energy, the system holds considerable promise for use in clinical cases. Other possibilities to extend this technique to clinical use are the miniaturization of the probes for intracavitary imaging with a mini-invasive approach and developing peroperative exploitation (37).

In conclusion, post-MI heart failure generates distinct and time-dependent trajectories of hypoxia in multiple organs and in the brain, detectable *in vivo* by using non-invasive and non-ionizing PAI technology. If further perfected, this emerging imaging modality could be applied to an array of clinical settings, and it could be used to evaluate early microvascular therapeutics.

## Data Availability Statement

The raw data supporting the conclusions of this article will be made available by the authors, without undue reservation.

## Ethics Statement

The animal study was reviewed and approved by Animal Care and Use Committee of the University of Montpellier (No. 13129-2018011910135259).

## Author Contributions

HD and PS planned the experiments and prepared the manuscript, performed statistical analysis and created manuscript figures. HD, AU, and PS performed the experiments and processed the data, processed and analyzed the imaging data. MP, NP, and EZ contributed to the experiments. HD, PG, PC, SR, NM, and PS edited the manuscript. All authors contributed to the article and approved the submitted version.

## Conflict of Interest

The authors declare that the research was conducted in the absence of any commercial or financial relationships that could be construed as a potential conflict of interest.

## Supplementary Material

Supplementary Figure 1 Evaluation of left ventricular ejection fraction **(A)** and heart rate **(B)** by high-resolution ultrasound in the sham and myocardial infarction (MI) groups at 4 h (*n* = 16 and 32 mice, respectively), day 1 (*n* = 15 and 27 mice, respectively), and day 7 (*n* = 8 and 16 mice, respectively) after surgery. ^**^*p* < 0.01; ^***^*p* < 0.001; sham vs. MI.

Supplementary Figure 2 Pearson correlation between anterior wall myocardial sO_2_ and anterior longitudinal strain **(A)** at 4 h (*n* = 38), **(B)** at day 1 (*n* = 36), and **(C)** at day 7 after surgery in the sham and myocardial infarction groups.

Supplementary Figure 3 Pearson correlation between anterior wall myocardial sO_2_ and left ventricular ejection fraction **(A)** at 4 h (*n* = 42), **(B)** at day 1 (*n* = 41), and **(C)** at day 7 (*n* = 24) after surgery in the sham and myocardial infarction groups.

Supplementary Figure 4 Pearson correlation between left ventricular ejection fraction and sO_2_ organs mapping **(A–C)** in the brain at 4 h **(A)** (*n* = 35), day 1 **(B)** (*n* = 32), and day 7 **(C)** (*n* = 13); **(D,E)** in the kidney at 4 h **(D)** (*n* = 35), day 1 **(E)** (*n* = 32), and day 7 **(F)** (*n* = 14), and **(G,H)** in the liver at 4 h **(G)** (*n* = 35), day 1 **(H)** (*n* = 32), and day 7 **(I)** (*n* = 14).

Supplementary Figure 5 Pearson correlation between global longitudinal strain and sO_2_ organ mapping **(A–C)** in the brain at 4 h **(A)** (*n* = 31), day 1 **(B)** (*n* = 26), and day 7 **(C)** (*n* = 12); **(D–F)** in the kidney at 4 h **(D)** (*n* = 31), day 1 **(E)** (*n* = 26), and day 7 **(F)** (*n* = 12) and **(G–I)** in the liver at 4 h **(G)** (*n* = 31), day 1 **(H)** (*n* = 26), and day 7 **(I)** (*n* = 12).

Supplementary Figure 6 Pearson correlation between lactate and sO_2_ organ mapping at 4 h post-myocardial infarction. **(A)** in the myocardial anterior wall, **(B)** in the brain, **(C)** in the kidney and **(D)** in the liver (*n* = 15).
