# Supplementary figures and images for: Experimental Myocardial Infarction Elicits Time-Dependent Patterns of Vascular Hypoxia in Peripheral Organs and in the Brain
Source: Front Cardiovasc Med. 2021 Jan 27;7:615507. doi: 10.3389/fcvm.2020.615507 (PMC7873295; doi:10.3389/fcvm.2020.615507)

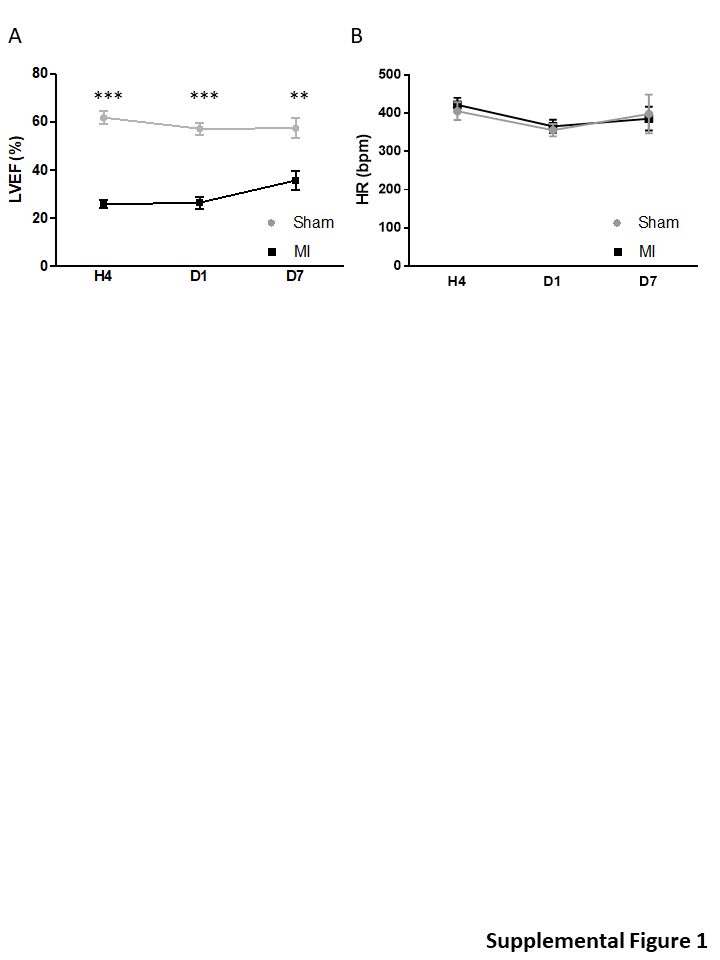

Supplement: Supplementary Figure 1 — Evaluation of left ventricular ejection fraction (A) and heart rate (B) by high-resolution ultrasound in the sham and myocardial infarction (MI) groups at 4 h (n = 16 and 32 mice, respectively), day 1 (n = 15 and 27 mice, respectively), and day 7 (n = 8 and 16 mice, respectively) after surgery. **p < 0.01; ***p < 0.001; sham vs. MI. [file Image_1.TIF]

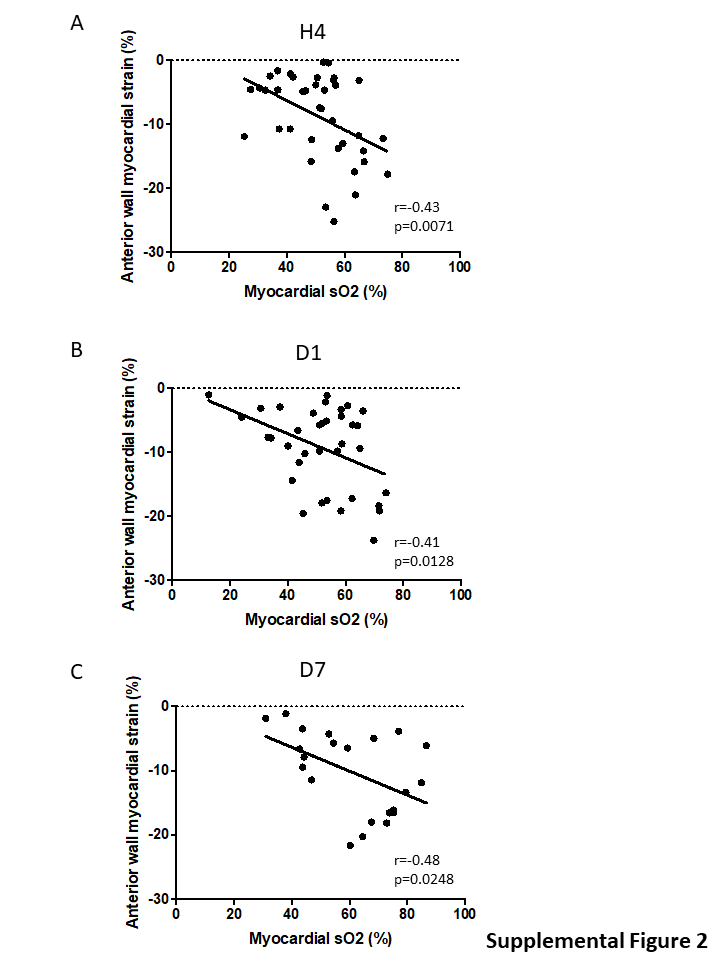

Supplement: Supplementary Figure 2 — Pearson correlation between anterior wall myocardial sO2 and anterior longitudinal strain (A) at 4 h (n = 38), (B) at day 1 (n = 36), and (C) at day 7 after surgery in the sham and myocardial infarction groups. [file Image_2.TIF]

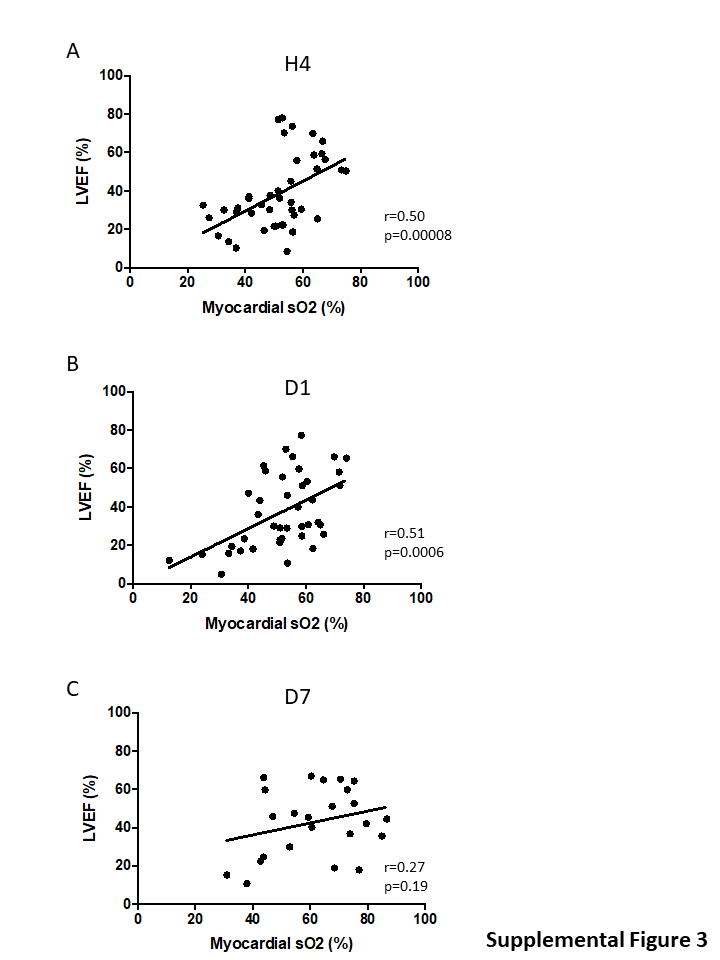

Supplement: Supplementary Figure 3 — Pearson correlation between anterior wall myocardial sO2 and left ventricular ejection fraction (A) at 4 h (n = 42), (B) at day 1 (n = 41), and (C) at day 7 (n = 24) after surgery in the sham and myocardial infarction groups. [file Image_3.TIF]

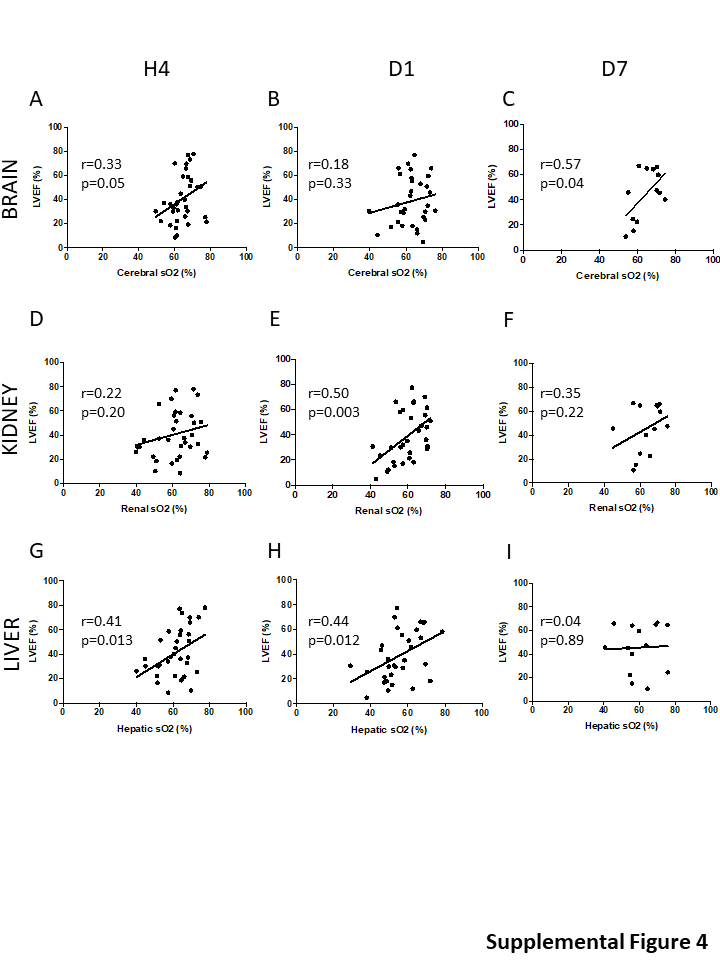

Supplement: Supplementary Figure 4 — Pearson correlation between left ventricular ejection fraction and sO2 organs mapping (A–C) in the brain at 4 h (A) (n = 35), day 1 (B) (n = 32), and day 7 (C) (n = 13); (D,E) in the kidney at 4 h (D) (n = 35), day 1 (E) (n = 32), and day 7 (F) (n = 14), and (G,H) in the liver at 4 h (G) (n = 35), day 1 (H) (n = 32), and day 7 (I) (n = 14). [file Image_4.TIF]

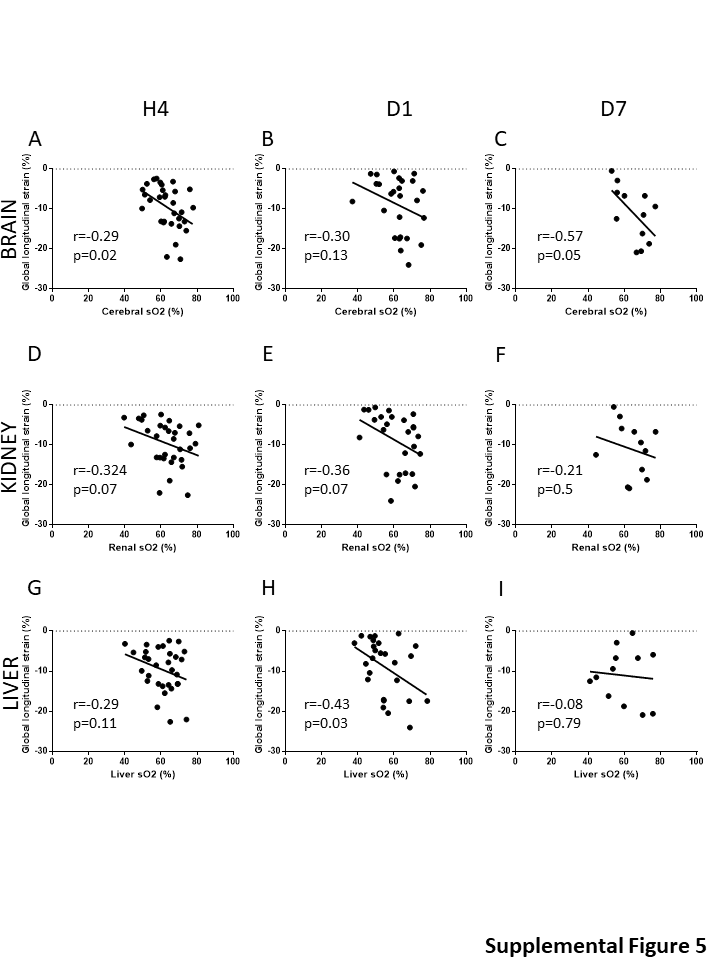

Supplement: Supplementary Figure 5 — Pearson correlation between global longitudinal strain and sO2 organ mapping (A–C) in the brain at 4 h (A) (n = 31), day 1 (B) (n = 26), and day 7 (C) (n = 12); (D–F) in the kidney at 4 h (D) (n = 31), day 1 (E) (n = 26), and day 7 (F) (n = 12) and (G–I) in the liver at 4 h (G) (n = 31), day 1 (H) (n = 26), and day 7 (I) (n = 12). [file Image_5.TIF]

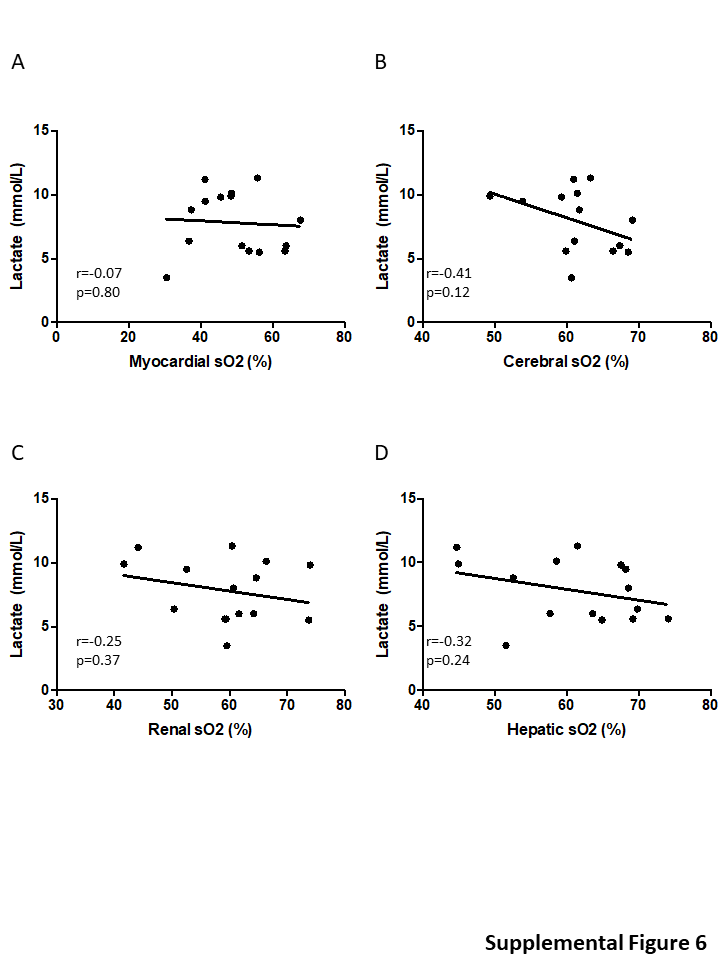

Supplement: Supplementary Figure 6 — Pearson correlation between lactate and sO2 organ mapping at 4 h post-myocardial infarction. (A) in the myocardial anterior wall, (B) in the brain, (C) in the kidney and (D) in the liver (n = 15). [file Image_6.TIF]
